# Changing gear: Experiences of how existing qualitative research can adapt to an unfolding health emergency

**DOI:** 10.3389/fsoc.2022.958861

**Published:** 2022-10-28

**Authors:** Theresa Jones, Luisa Enria, Shelley Lees, Mark Marchant, Olivia Tulloch

**Affiliations:** ^1^Anthrologica, Oxford, United Kingdom; ^2^London School of Hygiene and Tropical Medicine, London, United Kingdom

**Keywords:** qualitative research, long-term, health emergency, pivot, rapid

## Abstract

Long-term research projects are not always able to adapt to a new crisis and incorporate characteristics and approaches of rapid research to produce useful data quickly. Project AViD was a programme of research that ran between 2018 and 2022 to examine factors that shape vaccine confidence. The project initially focused on five country case studies looking at vaccines for Ebola, Measles, Rift Valley Fever and Zika. The COVID-19 pandemic emerged during this time and provided an opportunity to contribute to the pandemic's ‘million-dollar question'–how to deploy COVID-19 vaccines. Drawing on our experience as researchers, and specifically from AViD, we propose seven factors that can influence when and how longer-term qualitative research projects can adapt and contribute to the response to an unfolding health emergency. These include: (1) the phase of research in which the emergency hits; (2) the relative significance of the emergency in the research setting; (3) the specific methods and research team capacities; (4) existing operational links; (5) supportive ecosystems; (6) flexibility in research contracting and funding; and (7) the research team attitude and approach. We close with two considerations for longer-term research projects that find themselves having to “change gear” amid a public health emergency–the need to re-assess risks and benefits and the need to protect equitable partnerships.

## Introduction

When research projects are designed, protocols are written, a timeline is made, and the course is set. Not all projects have the flexibility to make significant changes. Researchers who find their planned work suddenly affected by a crisis, may ask themselves the question: do we swerve from the original course and respond to the crisis, or do we continue within the planned boundaries of our work? If we were studying measles in a community, and then Ebola cases were identified in the area, should we continue with our original focus? Who decides whether it is appropriate to shift focus? There are a multitude of considerations: methodological (e.g., safely adapting the research activities, developing new research questions and approaches); logistical (e.g., impact on travel); administrative (e.g., approval of funders to shift focus); and ethical (e.g., preserving the safety of participants and the research team, obtaining new approvals).

A strength of qualitative research is being, by nature, attuned to the wider context and iterative in design. As qualitative researchers, we look at experience, meaning and perspective, and wider social, cultural, political, and economic dynamics. Incorporating new and significant dynamics is part of our work. In comparison to certain quantitative research designs, qualitative research usually focuses on subjects and environments that are out of the control of the researcher (Robson and McCartan, 2016). As such, qualitative researchers must be able and ready to react to changes in the field and adapt their research design at any time (Edmondson and McManus, 2007).

Rapid qualitative research seeks to understand the impact of complex health emergencies by collecting and analyzing data within a short period of time (Beebe, 2014). The application of these approaches in emergencies has attracted much discussion (see, for example, Pink and Morgan, 2013; Beebe, 2014; Johnson and Vindrola-Padros, 2017; Vindrola-Padros et al., 2020). Characteristics and approaches common to rapid qualitative research are listed in Table 1.

**Table 1:** Characteristics and approaches of rapid qualitative research (Vindrola-Padros, 2021a).

**Characteristics of rapid qualitative research**	**Approaches of rapid qualitative research**
Iterative design, often carrying out data collection and analysis in parallel. Involve at least some degree of participatory research (including relevant stakeholders in the design and/or implementation of the study). Combine multiple methods of data collection and carry out triangulation during analysis. Can rely on the use of teams of researchers to cover more ground during data collection or contribute to data analysis. Are normally carried out within short study timeframes (a few weeks to a few months) or might include multiple data collection exercises of short duration (i.e. rapid but frequent feedback evaluations that run for a few years, but include short and intensive periods of data collection and analysis to share emerging findings as the evaluation is ongoing).	Bypassing the transcription of interview audio recordings to analyze data directly from the recordings. Reliance on interview or focus group notes instead of audio recordings and transcription. The use of techniques such as mind maps as focus groups are ongoing to summarize emerging findings. The implementation of structured observation guides to focus on the development of field notes during participant observation. The development of rapid data analysis techniques through the use of frameworks, tables or targeted coding techniques.

Longer-term research projects may have the advantage of additional time for rapport-building with participants and stakeholders, data collection, and analysis, and of being able to observe first-hand how beliefs and practices change over time. Yet, they are less suited to informing decisions with the urgency of rapid approaches and are often less action-oriented (Pink and Morgan, 2013). A small number of articles describe how longer-term research projects have been rapidly re-designed in time sensitive contexts to address an unfolding health emergency (Rahman et al., 2021; Vindrola-Padros and Johnson, 2021; Vindrola-Padros, 2021b). We know, anecdotally, that many more research projects pivoted during the COVID-19 crisis to address problems of the pandemic, but little has yet been written on this topic (Rahman et al., 2021). We therefore seek to address this knowledge gap regarding when and how longer-term qualitative research can pivot to respond to an unfolding health emergency and incorporate some of the characteristics and approaches of rapid research, to produce useful data quickly.

## AViD

Project AViD (Anthropological Exploration of Facilitators and Barriers to Vaccine Deployment and Administration During Disease Outbreaks) was a programme of research that ran between July 2018 to March 2022 to examine factors that shape vaccine confidence. At the inception of the project, five case studies were designed that would apply different qualitative and ethnographic methods across contexts in Sierra Leone, India, Uganda, and Brazil. These case studies would identify how vaccines can be optimally deployed during an outbreak in their respective settings.

The project originally focused on vaccines for Ebola, Measles, Rift Valley Fever and Zika. At the onset of the Ebola outbreak in the Equator region in the Democratic Republic of the Congo (DRC) in August 2018 additional funding was requested and approved to set up a sixth case study to explore the roll out of an Ebola vaccine. Then, when the COVID-19 pandemic emerged an extension of the AViD project into 2020 was requested and approved. As COVID-19 vaccines were rapidly developed, questions on how to deploy COVID-19 vaccines in low-income settings became the pandemic's “million dollar question”. The AViD research teams, drawing on our learning about other vaccines, started to question how we could contribute to COVID-19 vaccine deployment. Each of the case studies responded differently, which prompted the researchers to reflect on how they varied, and why.

These reflections were documented as part of a formal learning exercise which was embedded in the AViD project. The learning exercise ran in parallel to the roll-out of the case studies, with an external researcher (TJ) conducting quarterly interviews with the research team members, including the case study leads and national research team members of all the AViD projects. The primary aim was to provide opportunity for constructive reflection by the research teams on their work and to bring together information from across the case studies. Interview notes were coded and thematically analyzed. Emerging findings on the “lessons learned” were discussed with the wider AViD team in team meetings and workshops that were convened during the project. Conversations related to pivoting (or not pivoting) their research during the evolving COVID-19 crisis are documented below.

## AViD contributions to COVID-19 response

The Sierra Leone case study set out in 2018 to focus on political and economic factors influencing emergency vaccine deployment in Sierra Leone in the post 2014–2016 Ebola outbreak context. The project emerged from pre-existing collaborations in Kambia District during the Ebola vaccine trials, in which the case study lead had been working as a social scientist. The AViD research involved insider ethnography with a researcher embedded into the Kambia District Health Medical Team (DHMT), regular observations at the community and health facility level, power mapping workshops and key informant interviews. During the project, the team co-produced with the DHMT, a social science training package for Community Health Workers (CHW) to study vaccine confidence through a community-led ethnography approach (Enria, 2022). This emerged from early discussions with the DHMT where the need for evidence on vaccine confidence and access specific to the Districts' borderlands was identified as a priority by community engagement and vaccination leads. The case study was therefore designed to be flexible and responsive to the needs of the DHMT and for the data to support their priorities. This flexibility in design meant the project was well placed to adapt at the onset of the COVID-19 pandemic. In discussions with the DHMT as the pandemic emerged, District leadership decided it was important to develop rapid qualitative insights into unfolding events, based on previous experience of conducting research together on vaccine confidence and supply. The AViD research team therefore incorporated new topics into their research activities focusing on (i) rumors about COVID-19, (ii) the impacts of COVID-19 regulations on social economic activities and (iii) trust in the health sector. The team also obtained additional funding to allow the CHW work to pivot to focus on COVID-19, and they conducted observations on responses to COVID-19 in their villages. The urgent need for information required this long-term research to also adopt rapid research approaches (see Table 1), including the development of templates for rapid ethnography and rapid analysis techniques that could be quickly operationalised. Ongoing research had to be analyzed much faster to produce weekly briefings and slides to present at the District COVID-19 response meetings. Findings were shared in almost daily phone calls and collated into a briefing template according to the three major themes, alongside practical recommendations. Aside from offering these rapid insights, the team then also more slowly produced verbatim transcripts and longer-term analyses that complemented the rapid operational outputs.

The AViD case studies in Uganda, the DRC and India did not significantly pivot to the COVID-19 context. By the time of the escalation of the COVID-19 pandemic in mid-2020, these three case studies had already collected most of their data, and so this lens was not incorporated into their work. The Brazil case study originally focused only on Zika but faced some delays at project inception. This meant it could begin in 2020 with a focus on maternal vaccine confidence in Brazil in the context of Zika, dengue fever, chikungunya, and COVID-19.

In November 2021, 5 months before the close-out of the project, AViD made a further, significant pivot to contribute to the COVID-19 context. Prompted by the death of President Magufuli, the AViD project management proposed a sixth case study be added to the existing portfolio, in Tanzania. Given Tanzania's unique context of historically high vaccine confidence but emergent COVID-19 vaccine hesitancy following the former President Magufuli's COVID-19 denialism and rejection of vaccines, it was seen as an important opportunity to understand vaccine roll out. The case study leads equipped CHWs with basic social science research skills necessary to collect community-level data on knowledge, beliefs, rumors, and discussions related to COVID-19, prevention and control measures, vaccines and vaccine deployment. Document analysis and key informant interviews were conducted with COVID-19 response actors to identify strategy and policy areas that community-level findings could inform. To operate in a short timeframe, the research team used a number of rapid research approaches (see Table 1) including: multiple researchers collecting data (the CHWs), multiple data collection methods and triangulation, and diary notes instead of audio recordings and transcription. An iterative process for data collection and analysis was set up, whereby weekly reports from the CHWs were analyzed and further training was provided during data collection to explore in-depth key emergent themes. Given the need for information quickly, CHWs were in the field for a short duration. Table 2 summarises the AViD case study adaptations and their separate contributions to the COVID-19 response.

**Table 2:** AViD case studies adaptations to COVID-19.

**Country**	**Project adaptations**	**How findings informed COVID-19 response**
Brazil	Shift in disease of focus from Zika to COVID-19, maintaining focus on maternal vaccine confidence Remote data collection via phone surveys to protect participants and research team during early COVID-19 pandemic period	
Sierra Leone	Existing methods of community ethnography to understand views around vaccines and health services redirected to understanding views of COVID-19 response and vaccines Additional funds for community ethnography with CHWs using existing structure Shift from long term social science methods to rapid approaches to data collection, analysis and report-writing	Weekly presentations, phone calls and briefings provided to District Health Management Team (DHMT) Refinement of research methods to respond to DHMT learning interests Practical recommendations made to DHMT based on learning from ethnographic work
Tanzania	Development of a new AViD case study to replace other research previously stalled by President John Magufuli Research protocols on community views of clinical trials amended to focus on perspectives of COVID-19 disease and vaccines Community health workers trained to document rumors on COVID-19 effects on livelihoods in 2020 instead documented rumors on vaccines after delay of previous project Inclusion of Ministry of Health Department of Risk Coordination and Community Engagement in study design Addition of data collection site in region of interest to Ministry of Health	Findings and recommendations shared with Ministry of Health Department of Risk Coordination and Community Engagement Findings used to develop, test and share with Ministry of Health a responsive training package on COVID-19 vaccines for CHWs Developed community engagement activities for region with low uptake of COVID-19 vaccines

Beyond the individual case studies, the AViD research team as a collective also made several contributions to the global COVID-19 response, in terms of publishing operational guidance and other materials, which are detailed further below.

## What factors influence this changing of gears?

Drawing on the experience of the AViD project – particularly the Sierra Leone and Tanzania case studies, and on other relevant research, including by Johnson and Vindrola-Padros (2017), Vindrola-Padros (2021b), Vindrola-Padros and Johnson (2021), and Rahman et al. (2021)—we propose seven factors that influence when and how longer-term qualitative research can adapt and contribute learning to an unfolding health emergency.

In this article, we focus particularly on the experiences of the Sierra Leonean and Tanzanian case studies so as to be able to discuss the complexities of “changing gears” in more depth. These two case studies encountered different challenges that allowed us to draw out some key reflections, particularly on the significance of building long-term partnerships to facilitate short-term project adaptations and the contextual specificity of political sensitivities around conducting crisis research.

1. **Timing—the phase of the research when the emergency hits** The earlier in the research process that the crisis hits, the more significantly a project can re-focus on the emergency. The AViD case studies all progressed at different rates, and so were intersected by COVID-19 at different phases. The Tanzania case study began when the pandemic was well underway, and so was able to fully focus on COVID-19. Being part of a larger ongoing project allowed this piece of work to get off the ground and begin collecting data rapidly.
  Being able to produce findings at the time they are needed is also important for long-term research to contribute to a crisis. Vindrola-Padros and Johnson (2021) identify the importance of timing the generation of research findings to inform decision-making processes. The AViD researchers as a collective published operational guidance on vaccine trials in October 2020 to coincide with the Phase 3 trials of the first COVID-19 vaccine candidates (Burns et al., 2020). As soon as Tanzania was politically able to focus on COVID-19 vaccination, an existing research collaboration between government research institutions and the MoH ensured that the Tanzania case study was able to quickly get ethical approval, activate training and deployment of CHWs as well as recruit local social scientists.
2. **The relative significance of the emergency in the research setting** In contexts with other pressing priorities, and insufficient resources to act differently, long-term research cannot substantially pivot to focus on an unfolding health emergency. The COVID-19 pandemic has been experienced differently across the globe, both in its actual health impacts, but also in the relativity of it as a threat. COVID-19 may have only partially altered life in countries already ridden with other priority issues, for example areas with active conflict (Bond et al., 2020). The AVID case study in Sierra Leone was well placed to contribute more significantly to a COVID-19 response. However, over time, as reported cases drastically diminished and the health service continued to be over-stretched across different priorities with limited funds, the demand for COVID-19-related research fell.
  Political sway is also an important determinant of whether ongoing research can contribute learning to an emerging crisis. During the presidency of John Magufuli, in the early days of the pandemic in Tanzania, COVID-19-related research was not given approval. By early 2021 the president not only denied the existence of COVID-19 in Tanzania but also questioned the efficacy of COVID-19 vaccines and therapeutics that had been developed in high income contexts. During this time public health officials worked as best they could to instill public health measures despite being unable to report cases. Following his death in March 2021, a moratorium on discussing COVID-19 was lifted and public health measures were put into place and the COVID-19 vaccination campaigns began. Support at government level for the new AViD case study became possible (see Lees et al., 2022).
3. **Methods and research team capacities** The methods used in a long-term research project can determine whether pivoting to focus on an emerging crisis is possible. Some qualitative research methods and approaches lend themselves better than others to being responsive in a crisis. The AViD Sierra Leone case study included an insider ethnographer embedded into the DHMT, whose other role was as a field epidemiologist. Being embedded into an operational team with clear links to any emergency response, made understanding and contributing to a new health crisis possible, although with some limitations, as noted above. Johnson and Vindrola-Padros (2017) similarly describes how being embedded into the UNICEF vaccination team in Pakistan allowed the researcher to re-focus on COVID-19 using a gendered approach. Additionally, response to COVID-19—and all disease outbreaks—demands an interdisciplinary approach, and the design of the Sierra Leone case study meant that epidemiologists, case management teams and social scientists were already working together.
  The Sierra Leone and Tanzania AViD case studies both used video conferencing and social media as part of their data collection and supervision methods, which worked well in an emergency context and infectious disease outbreak. Without these methods COVID-19 travel restrictions and other public health and social measures (e.g., physical distancing) would have otherwise made data collection impossible. Rahman et al. (2021) describe the unique value of video conferencing software, including how fruitful the “chat box” can be as part of observation work. However, they conclude that not every qualitative method can become effectively virtual, for example, their projects that relied on participatory methods were more difficult to effectively move online. They also warned that online research can also further disenfranchise marginalized groups, who may not have access to it or know how to use it (Sevelius et al., 2020).
  If longer-term research initiatives can adapt to understanding issues *as they evolve*, they can be more helpful in an emergency setting. Vindrola-Padros and Johnson (2021) emphasize the need to use innovative data collection and analysis methods to understand and address the evolving issues during a crisis. They suggest running data analysis in parallel to collecting data to produce findings in “real-time”. Both the Sierra Leone and Tanzania AViD case studies shifted to work with their COVID-19 related data in this way, rather than first collecting and *then* analyzing data, the trajectory of most longer-term research models. In Sierra Leone, COVID-19 data was shared within the team daily, including sharing reports, WhatsApp messages and ethnographic observations, bringing together insights from the insider ethnography and the CHWs' work in their communities. In a weekly call, these findings were discussed to clarify their significance and to agree on recommendations.
  It must be acknowledged that producing rapid findings comes with trade-offs in terms of depth and ability to trace changes over time. However, in the AViD project we were in the fortunate position of being able to feed findings back rapidly whilst also having the capacity to do more formal, “slower” analysis of transcripts and ethnographic observations over the 4 years of the project. This may not always be possible, and as such the trade-offs need to be recognized and their potential consequences considered.
4. **Existing operational links** Accessing windows of opportunity to influence crisis programming and policy is particularly important for a longer-term research project to contribute to a public health emergency. Although not established as emergency response research, the AViD Sierra Leone case study was able to contribute to the COVID-19 response at a district-level. Having worked in the Kambia district previously for several years, the Sierra Leone research lead had built long standing relationships with the DHMT and other important health system actors who mobilize during crises, including healthcare workers and CHWs in various communities across the district, this meant that research findings could be delivered directly to those who were able to act on them. These long-term partnerships and relationships with affected communities was a strength, making it easier to conduct rapid research that engaged in ethical and sensitive ways in a moment of crisis. The research on COVID-19 was designed collaboratively with these existing partners and developed organically from these existing relations, rather than requiring new working relationships to be formed at a time of extreme vulnerability.
  The AViD Tanzania case study also had strong operational links with the COVID-19 response. The case study leads mobilized an existing collaboration between LSHTM, the National Institute for Medical Research (NIMR) and the Ifakara Health Institute (IHI), which intended to explore deliberative engagement in clinical trials. The co-investigator was the co-chair of the Risk Communication and Community Engagement (RCCE) pillar of the COVID-19 response. This meant there were existing connections with the Ministry of Health, Community Development, Gender, Elderly and Children (MoHCDGEC) Health Promotion department before the research began. As a result, relevant parts of the social science training package (including reflective listening and emotional intelligence) were integrated into national Tanzanian CHW training curriculum on COVID-19.
  The AViD project researchers as a collective also made use of their relationships with platforms such as the Social Science in Humanitarian Action Platform (SSHAP) to host and disseminate operational guidance resources including “Clinical and Vaccine Trials for COVID-19: Lessons Learned from Social Science” (Burns et al., 2020), “Social Science Research for Vaccine Deployment in Epidemic Outbreaks” (Bowmer et al., 2020) and “Citizen ethnography in Outbreak Response: Guidance for Establishing Networks of Researchers” (Enria, 2022).
5. **Supportive ecosystems** For longer-term research to contribute to an unfolding health emergency, certain systems and structures are needed: (1) to create demand for qualitative research on the crisis and (2) for these findings to be taken up. In Kambia, Sierra Leone, there was a weekly District COVID-19 Response meeting, in which the AViD research team shared their key findings and recommendations. These briefings were specifically geared to informing operations. For example, one presentation highlighted the research finding around the mistrust of “strangers” who had been sent to communities for community engagement. This was then addressed through a re-organization of teams deployed to the field. In Tanzania, there had been no formal COVID-19 response structures under Magufuli. These systems were created after his death, and research about vaccines became possible. The AViD research team embedded itself from the beginning into the RCCE pillar, ensuring there was an audience for the research findings.
  Other ecosystem factors which enabled AViD to contribute to the COVID-19 response included decision-makers who understood the value of qualitative research, having “champions” of the work amongst decision-makers and having adequate funding to establish these structures and for decision-makers to act on findings of the research.
  In addition to emergency response infrastructure, higher education and research institutions must be supportive, look at the context and adjust their expectations accordingly for ongoing research to be able to adapt and contribute new learning to a public health emergency, (Rahman et al., 2021). The AViD Tanzania case study experienced delays because they chose to amend an existing ethical approval submission rather than submit a new application. In emergencies, social science researchers often rely on expedited ethical review processes, wherein proposals for research deemed as having minimal harm to participants are reviewed in a matter of days rather than weeks. The Tanzania team found out it was not possible to expedite an amendment to an existing submission with the Tanzanian National Health Research Ethics Committee. Ethical review processes that allow for amendments to existing submissions to also be expedited could facilitate adaptations during emergencies.
6. **Flexibility in research contracting and funding** Having a research funder who allows for topics and timelines to shift in light of an evolving health emergency is necessary for longer-term research to adapt and contribute new learning. The funder of AViD was very supportive of shifting the research focus, where possible, to COVID-19. This was a welcome and positive position because during the pandemic research funders who wanted rapid qualitative research to inform clinical decision-making or to provide evidence for public health policy did not tend to think of also requesting adaptions to existing studies. In addition to extending the timeline of the project, the AViD funder also allowed the additional Tanzania case study to be introduced at a late stage of the project. The importance of donor flexibility also contributed to the “resilient” and adaptive research described by Rahman et al. (2021) who received a no-cost extension to continue part of their research and to bring in a new COVID-19-specific angle.
  One hypothetical risk raised by the AViD research team was the scenario whereby funders request a substantial refocus on an emerging crisis even when the original focus of the research retains its importance. This could result in the original research topic being neglected. In this case, they argue it is important for researchers to communicate this clearly with project funders.
7. **Research team approach and attitude** The approach and even the attitude of the research team may play a role in how longer-term research can contribute to an unfolding health emergency. One AViD researcher described how, at times, “*we limit ourselves”* to focusing on what we know, whereby reacting to an unknown crisis may require a degree of flexibility and boldness. Researchers may identify as “long-term researchers” and may not see their role in a crisis setting. Despite this point, AViD researchers did reflect that many of their network of research colleagues had been eager to contribute to the COVID-19 pandemic however they could. During this pandemic, almost everyone operating in a health setting was expected to adapt and contribute to the pandemic response. So, even for those researchers who paused their research projects, this may have brought about a change in attitude in terms of whether they are “crisis researchers” or not.
  Critical reflection on research priorities has helped other researchers adapt their research projects during COVID-19 (see Rahman et al., 2021). The learning exercise embedded within the AViD project was one forum for the research teams to consider if and how to adapt their work, in addition to regular project calls.

## Key considerations

Continuing long-term research during an unfolding health emergency, whether or not the research is able to contribute directly to that crisis, introduces significant new dynamics into the research. Learning from the diverse experiences of the AViD project, we highlight two key considerations for researchers.

### Re-assess risks and benefits

During an emerging crisis, the first question that researchers should ask themselves from an ethical standpoint is ‘should we be carrying out research *at all* at this time?' (Vindrola-Padros et al., 2020; Rahman et al., 2021). This should include considerations of whether the research team are best placed to conduct research on the health emergency as it unfolds and whether this re-focusing contributes to a distortion of priorities, whereby other issues get neglected because everyone is shifting focus to the emergency. Accuracy and quality are also ethical requirements, and as such it is important to assess research teams' ability to protect the integrity of the research when it shifts to a rapid approach. As noted in this article, there may be some inevitable trade-offs. For example, rapid analysis for operational purposes may make it difficult to capture nuance and change that can be observed through long-term analysis and reflection. It is advisable to complement rapid, operational research with longer-term, careful analysis of research findings that can contribute to reflections in “peace time” to support future crisis planning.

Continuing long-term research *at all* during an emerging crisis requires that risks and benefits be re-evaluated. Research itself should not exacerbate any risks that the crisis has amplified—for example, the risk of transmitting infection during data collection, with the researcher themselves a potential “*vector of transmission”*(Bond et al., 2020). Research should not be an additional burden on participants who may already be under enormous pressure related to the crisis (Rahman et al., 2021). Benefits for participants may also change, for example the research may enable participants to ask questions and voice concerns about the nature of the crisis in question.

Any official public health measures must be followed. A deep understanding of the specific setting can help to identify any localized norms around these protective measures and any other context-specific considerations. For example, members of the AViD Sierra Leone team had previously worked during the 2014–16 Ebola response, including as contact tracers and as social scientists in an Ebola vaccine trial. They were aware of how those memories might affect communities' responses to COVID-19 and were able to rapidly shift to research that considered localized protective measures. For example, the team monitored the development of community responses such as chiefdom task forces that emerged from learnings during Ebola to determine whether they needed to be engaged in the research process.

Rather than continuing and minimizing danger, the most ethical response by researchers may be postponing altogether. However, putting research on hold may have its own ethical implications, in terms of responsibilities to participants and time-sensitive data (Wood et al., 2020).

### Protect equitable partnerships

The COVID-19 pandemic transformed and intensified AViD's transnational institutional collaborations and partnerships. Restricted foreign travel meant that AViD depended more heavily on in-country research team members. There is a long history of unequal partnerships and research collaborations between Global South and Western research institutions (Boum et al., 2018). Remote research has the potential to widen power inequalities when local researchers take on more risk of direct field work during a disease outbreak. Dunia et al. (2020) suggests that post-COVID 19 research institutions, funding agencies and ethics boards need to ask more questions about the role of “facilitating” vs. “contracting” researchers at various stages of research in terms of safety and risk implicit in each person's role. Where remote working or other restrictions changes these roles, this must be re-analyzed.

As AViD researchers, we were keenly aware of the power imbalances that continue to dominate Western-led and funded research. It is difficult to shift such systemic inequities and existing research structures often reproduce them. The project endeavored to engage reflexively with these dynamics, and it offered opportunities to further strengthen international relationships and to support existing social science capacity and leadership in country, contributing to a “*social science legacy”*. Congolese, Sierra Leonean, Tanzanian and Ugandan researchers contributed their expertise to the development of the different methods and engagement activities, in addition to supporting data collection and analysis. COVID-19 highlighted both the possibilities of remote mentorship for locally-led research but also the operational apparatus, such as institutional contracting or financing systems, that can reinforce barriers to power sharing in transnational research partnerships, especially when a crisis emerges. For example, some of the AViD case studies found it difficult to re-channel money and make international payments quickly when they adapted activities in light of COVID-19. Learnings from the AViD project included to formally partner from the outset of the funding application with public health partners, rather than sub-contracting, and to ensure flexibility in financing systems to support collaborative and responsive research.

Concerns about shifting risks onto local researchers in the Sierra Leone case study led to the development of adaptive protocols—such as the CHWs writing ‘lockdown diaries' when movement was restricted—that could be activated by the researchers to align to local regulations and their own risk assessments. At some points, during different waves of the pandemic and as its trajectory was uncertain, the AViD research teams halted their activities altogether.

## Conclusion

Researching in crisis gives rise to unique ethical, political, and practical challenges that researchers of conflict and health emergencies have engaged for years. We have highlighted how long-term qualitative research, with its focus on context, is uniquely positioned to provide relevant insights for rapid response to public health emergencies. In this article we explored a component of crisis research that has been relatively unexplored: should we as researchers “change gear” to respond to an emergent crisis and, if so, what factors facilitate this shift? Drawing on experiences from the AViD project and particularly its case studies in Sierra Leone and Tanzania, we have highlighted several factors that could be relevant for researchers pivoting to work on an unforeseen crisis. These include questions of timing, the relative ability and willingness of local emergency responses to take up research findings, existing research and operational links and the flexibility of research funding to be able to adapt. These practical considerations are underpinned by ethical questions which ought to be further explored, including questions about shifting risk, the impact of emergencies on global research architectures and their associated power dynamics and whether research on crisis is always desirable. Our case studies highlight significant practical challenges but also shed light on the possibilities that emerge when existing relationships give rise to organic demand for research that supports crisis response.

## Author contributions

All authors contributed to the research described, including the lessons learned activity. All authors contributed to the article and approved the submitted version.

## Funding

AViD was funded by NIHR.

## Conflict of interest

The authors declare that the research was conducted in the absence of any commercial or financial relationships that could be construed as a potential conflict of interest.

## Publisher's note

All claims expressed in this article are solely those of the authors and do not necessarily represent those of their affiliated organizations, or those of the publisher, the editors and the reviewers. Any product that may be evaluated in this article, or claim that may be made by its manufacturer, is not guaranteed or endorsed by the publisher.

## References

[B1] Beebe J. (2014). Rapid Qualitative Inquiry: A Field Guide to Team-Based Assessment (2nd ed.). Rowman & Littlefield.

[B2] Bond K. D. Lake M. Parkinson S. E. (2020). Lessons from Conflict Studies on Research during the Coronvirus Pandemic. SSRC. Available online at: https://items.ssrc.org/covid-19-and-the-social-sciences/social-research-and-insecurity/lessons-from-conflict-studies-on-research-during-the-coronavirus-pandemic/ (accessed November 2020).

[B3] Boum I. I. Y. Burns B. F. Siedner M. Mburu Y. Bukusi E.. (2018). Advancing equitable global health research partnerships in Africa. BMJ Global Health. 3, e000868. 10.1136/bmjgh-2018-000868 30167335 PMC6112391

[B4] Bowmer A. Lees S. Marchant M. (2020). Social Science Research for Vaccine Deployment in Epidemic Outbreaks. Social Science in Humanitarian Action Platform. Available online at: https://www.socialscienceinaction.org/resources/social-science-research-for-vaccine-deployment-in-epidemic-outbreaks/ (accessed November 2020).

[B5] Burns R. Enria L. Bowmer A. Vanderslott S. Lees S. (2020). “Clinical and Vaccine Trials for COVID-19: Lessons Learned from Social Science,” in Social Science in Humanitarian Action Platform. Available online at: https://www.socialscienceinaction.org/resources/clinical-and-vaccine-trials-for-covid-19-key-considerations-from-social-science/ (accessed January 2021).

[B6] Dunia O. A. Baaz E. M. Mwambari D. Parashar S. Toppo A. O. M.. (2020). The COVID-19 Opportunity: Creating more Ethical and Sustainable Research Practices. SSRC. Available online at: https://items.ssrc.org/covid-19-and-the-social-sciences/social-research-and-insecurity/the-covid-19-opportunity-creating-more-ethical-and-sustainable-research-practices/

[B7] Edmondson A. M. McManus S. E. (2007). Methodological fit in management field research. Acad Manage Rev. 32, 1155–1179. 10.5465/amr.2007.26586086

[B8] Enria L. (2022). “Citizen ethnography in Outbreak Response: Guidance for Establishing Networks of Researchers,” Social Science in Humanitarian Action Platform (SSHAP). Citizen Ethnography in Outbreak Response: Guidance for Establishing Networks of Researchers - Social Science in Humanitarian Action Platform (socialscienceinaction.org)

[B9] Johnson G. Vindrola-Padros C. (2017). Rapid qualitative research methods during complex health emergencies: a systematic review of the literature. Soc. Sci. Med. 189, 63–75. 10.1016/j.socscimed.2017.07.029 28787628

[B10] Lees S. Enria L. James M. (2022). Contesting the crisis narrative: epidemic accounts in Sierra Leone, democratic Republic of Congo, and Tanzania. Disasters. 10.1111/disa.12535 35348243 PMC10091748

[B11] Pink S. Morgan J. (2013). Short term ethnography: intense routes to knowing. Symbolic Interact. 36, 351–361. 10.1002/symb.66

[B12] Rahman S. A. Tuckerman L. Vorley T. Gherhes C. (2021). Resilient research in the field: Insights and lessons from adapting qualitative research projects during the covid-19 pandemic. Int. J. Qual. Method. 20. 10.1177/16094069211016106

[B13] Robson C. McCartan K. (2016). Real World Research. New York, NY: John Wiley & Sons.

[B14] Sevelius J. Gutierrez-Mock L. Zamudio-Haas S. McCree B. Ngo A. Jackson A.. (2020). Research with marginalized communities: challenges to continuity during the COVID-19 pandemic. Aids and Behavi. 24, 2009–2012. 10.1007/s10461-020-02920-3 32415617 PMC7228861

[B15] Vindrola-Padros C. (2021a). Doing Rapid Qualitative Research. New York, NY: Sage Publishing.

[B16] Vindrola-Padros C. (2021b). Rapid Ethnographies: A Practical Guide. London: University College London. 10.1017/9781108623568

[B17] Vindrola-Padros C. Chisnall G. Cooper S. Dowrick A. Djelloui N. Symmons S. M.. (2020). Carrying out rapid qualitative research during a pandemic: emerging lessons from COVID-19. Qual. Health Res. 30, 2192–2204. 10.1177/1049732320951526 32865149 PMC7649912

[B18] Vindrola-Padros C. Johnson J. (2021). “The use of rapid qualitative research in time-sensitive contexts,” in: Temporality in Qualitative Inquiry, Clift B. C. Gore J. Gustafsson S. Bekker A. Batlle I. C.. (eds). Routledge. 10.4324/9781003083504-11

[B19] Wood E. S. Rogers D. Sivaramakrishnan K. Almeling R. (2020). Resuming Field Research in Pandemic Times. SSRC. Available online at: https://items.ssrc.org/covid-19-and-the-social-sciences/social-research-and-insecurity/resuming-field-research-in-pandemic-times/

